# Supplementary figures and images for: Sex-Specific Differences in Cardiovascular Risk, Risk Factors and Risk Management in the Peripheral Arterial Disease Population
Source: Diagnostics (Basel). 2022 Mar 25;12(4):808. doi: 10.3390/diagnostics12040808 (PMC9027979; doi:10.3390/diagnostics12040808)

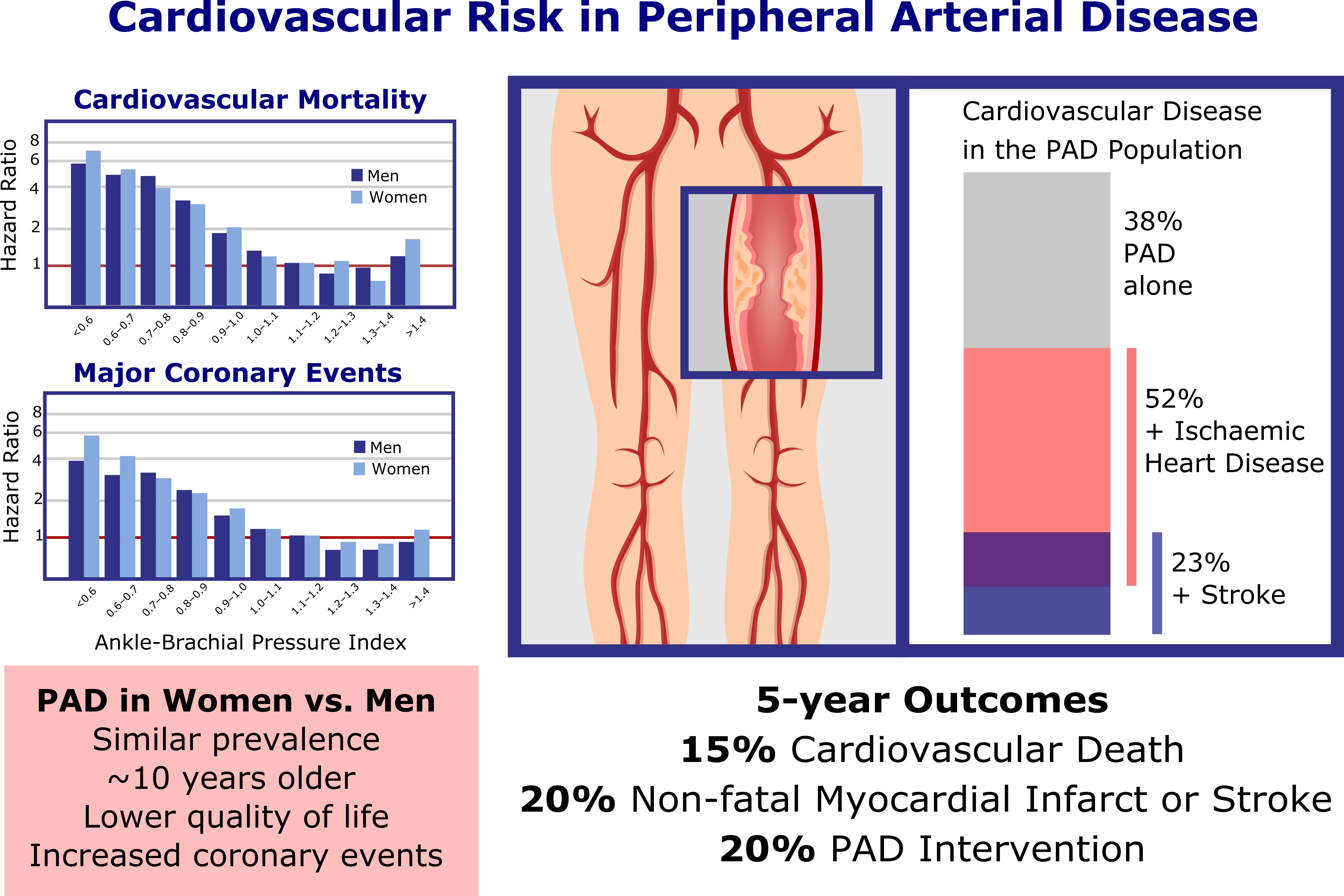

Supplement: Supplementary file 1 [file diagnostics-12-00808-s001.zip › diagnostics-1629696-supplementary/Figure S1.png]

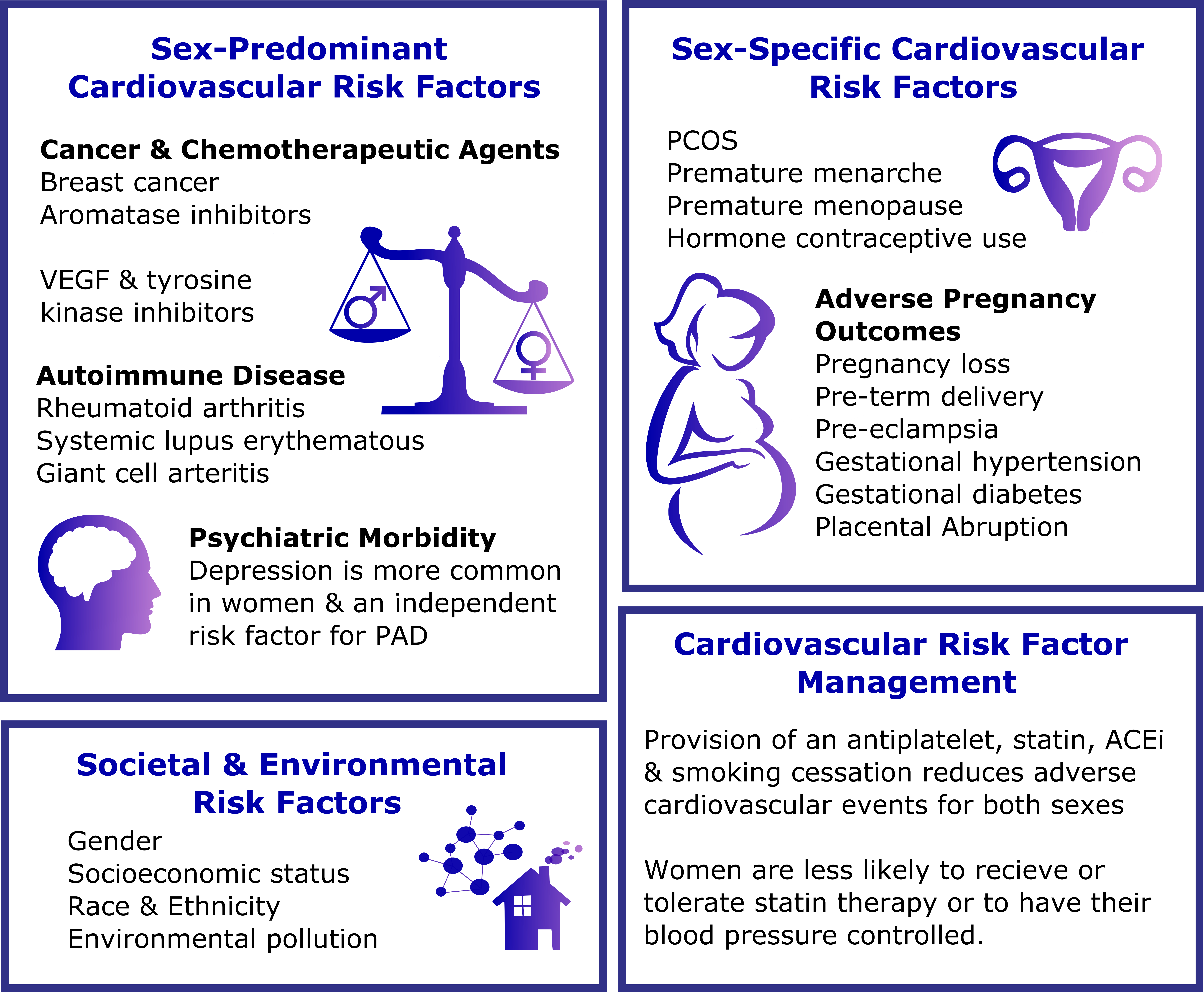

Supplement: Supplementary file 1 [file diagnostics-12-00808-s001.zip › diagnostics-1629696-supplementary/Figure S2.png]
